# The dual role for probiotics use in dental practices

**DOI:** 10.3389/froh.2023.1336565

**Published:** 2023-12-21

**Authors:** Debra A. Goff, Lynne V. McFarland, Stuart Johnson, Douglas W. Goff

**Affiliations:** ^1^ Global Antibiotic Stewardship, The Ohio State University Wexner Medical Center, Columbus, OH, United States; ^2^ Department of Pharmacy, The Ohio State University College of Pharmacy, Columbus, OH, United States; ^3^ Public Health Reserve Corp, Seattle, WA, United States; ^4^ Infectious Diseases Division, Loyola University Medical Center, Chicago, IL, United States; ^5^ Departments of Medicine and Research, Edward Hines Jr. VA Hospital, Hines, IL, United States; ^6^ Gilbert and Goff Prosthodontists, Upper Arlington, Columbus, OH, United States

**Keywords:** antibiotics, dental infections, antibiotic-associated diarrhea, *Clostridioides difficile*, probiotics

## Introduction

Antibiotics are commonly prescribed as prophylaxis for dental procedures and for treatment of dental infections. Although antibiotics have an important role in preventing and treating infections, antibiotics may have unintended consequences when they disrupt the oral and intestinal microbiome of the patient (1). The consequences of antibiotic use may include a lengthy disruption (lasting 2–6 months) of the protective microbiome, leading to increased risk of infection by opportunistic pathogens (such as *Streptococcus mutans* or *Clostridioides difficile*), development of antibiotic-associated diarrhea (AAD), development of antibiotic-resistant bacteria, and the development of adverse reactions ranging from mild rash to serious reactions requiring hospitalization (2–5). AAD occurs in 10%–40% of patients receiving antibiotics (15% in outpatients) and may last 1–22 days (2). AAD caused by *C. difficile* ranges from mild diarrhea to severe pseudomembranous colitis and may result in hospitalization and death (2). Probiotics may have three potential roles in dental practices: (1) the prevention of antibiotic-associated complications, (2) prevention of dental caries, or (3) treatment of periodontal disease. The interest in probiotics in dental practices has been growing, but there is no current consensus on how probiotics can best be utilized (6, 7). Our aims are to discuss the role of probiotics when used to limit complications of antibiotics and to prevent or treat dental infections.

### Probiotics to prevent antibiotic-associated consequences

Probiotics are living microbes which have proven health benefits and typically are well-tolerated. Probiotics may have multiple mechanisms prevent antibiotic-associated diarrhea: restoration of the normally protective microbiome, interference with pathogen attachment to oral or intestinal cells, and regulating immune responses (8–11). Recent guidelines from the American Gastroenterology Association (AGA) recommend four types of probiotics for the prevention of *C. difficile* infections: *Saccharomyces boulardii* CNCM I-745 (Floratsor™), a three-strain mixture of *Lactobacillus acidophilus* CL1285, *L. casei* LBC80R, *L. rhamnosus* CLR2 (Bio-K+™), another three-strain mixture of *L. acidophilus*, *L. bulgaricus* and *Bifidobacterium bifidum* and a four-strain mixture of *L. acidophilus*, *L. bulgaricus, Bifidobacterium bifidum* and *Streptococcus thermophilus* (12). However, recommendations for the last two of the probiotic mixtures were based on only one trial and only meeting abstracts were published, thus the evidence is weak. Another review found strong evidence for two probiotics [*L. rhamnosus* GG (Culturelle™) and *S. boulardii* CNCM I-745 (Florastor™)] to prevent AAD (13). Typically the probiotic was started within 24 h of the antibiotic and continued while the patient was on antibiotics, then given for five-seven days afterwards to allow the normally protective microbiome to recover (14). It should be noted that not all “probiotic” strains or mixtures are equally effective to prevent these complications. The efficacy of probiotics has been determined to be both strain-specific and disease-specific, thus conclusions on which probiotic should be recommended to prevent different complications such as AAD or *C. difficile* infections, etc. need to be based on evidence done with specific strains (15). Often the type of probiotic that is available in stores or online are not the appropriate type of probiotic that is needed to prevent these complications. Based on the published evidence, several probiotics can be recommended for the prevention of AAD and CDI, as shown in the Table 1.

**Table 1:** Levels of recommendations for probiotic products for antibiotic-associated diarrhea, *C. difficile* infections and periodontal disease.

Brand name	Probiotic strain(s)	Dosage per day	Prevent AAD	Prevent CDI	Prevent dental caries	Treat PD
Florastor	*S. boulardii* CNCM-745	2–4 capsules	I	I	Nd	Nd
Bio-K+	*L. acidophilus* CL1285, *L. casei* LBC80R, *L. rhamnosus* CLR2	1–2 capsules or 1 bottle	I	II	Nd	Nd
Yakult	*L. casei* Shirota	10 ml milk	Nd	Nd	III	Nd
Darolac	*L. helveticus* R52 + *L. rhamnosus* R11 + *Bifido. longum* R175 + *S. cerevisiae boulardii* CNCM I-1079	10 ml mouthwash or 1–2 grams in liquid	Nd	Nd	III	I
Prodentis	*L. reuteri* ATCC 17930 + *L. reuteri* PTA 5285	1–2 lozenges	Nd	Nd	Nd	I

Level of recommendation: I, evidence from at least two RCTs (highest level); II, evidence from cohort or quasi-experimental facility level clinical studies (moderate); III, evidence from only one RCT (low).

AAD, antibiotic-associated diarrhea; Bifido., *Bifidobacterium*; CDI, *Clostridioides difficile* infection; L., *Lactobacillus*; ml, milliliters; Nd, not done; S., *Saccharomyces*; PD, periodontal disease; RCTs, randomized controlled trials.

### Probiotics to prevent dental caries

One mechanism to prevent the development of dental caries involves reducing the number of carigenic bacteria, such as *Streptococccus mutans*. A review of 17 different types of probiotics found 14/19 of the trials reduced the counts of *Strept. mutans* after probiotic use, but many types were supported by only one trial (16). Several types of probiotics may show promise in reducing caries by this mechanism: *L. casei* Shirota (17) or a 3-strain probiotic blend of *L. rhamnosus* R011 *Bifidobacterium longum* R175 and *Saccharomyces cerevisiae boulardii* CNCM I-1077 (Sporolac™) (18) or a four-strain blend (Durolac ™), which added *L. helveticus* R052 to the three strains in Sporolac (19).

### Probiotics to treat dental infections

Another role for probiotics may be for the treatment of dental infections. Several reviews and meta-analyses assessed randomized, controlled trials of different probiotics for periodontal diseases and found promising results with *Lactobacilli* probiotics, but were unable to identify which specific *Lactobacilli* strains are the most effective (20–22). A meta-analysis of 36 randomized, controlled trials found 15 of 17 (88%) of trials with a two-strained probiotic (*L. reuteri* ATCC 17930 and *L. reuteri* PTA5289) significantly improved chronic periodontitis symptoms, but other types of probiotics (*L. rhamnosus* SP-1, *L. rhamnosus* DSM14869, *L. brevis* CD2, etc.) were not effective (23). The mechanisms of probiotics for the treatment of periodontal infections involve the interference with pathogen attachment of mucosal surfaces, production of antibacterial bacterocins and reducing pro-inflammatory cytokine production (20, 23–25). The ability of probiotic strains to reduce periodontal pathogens, such as *Porphyromonas gingivalis*, *Aggregatibacter actinomycetemcomitans, Prevotella intermedia* or *Fusobacterium nucleatum* has not be well studied (26). A two-strain mixture of *L. reuteri* 17930 and *L. reuteri* PTA5285 (24) and a four-strain mixture of *L. helveticus* R52 + *L. rhamnosus* R11 + *Bifido. longum* R175 + *S. cerevisiae boulardii* CNCM I-1079 (27, 28) have shown potential for treating periodontal disease, as shown in Table 1. The effect of probiotics for the treatment of dental infections may fade after probiotics are discontinued, thus further research into not only which probiotic strains are most effective, but the optimum duration and dose is needed.

Dentists may consider recommending probiotics to their patients who require antibiotics and may be at higher risk of developing antibiotic-associated complications, such as AAD or CDI. Risk factors for CDI include age >65, use of proton pump inhibitors, antibiotics, immune-compromised or hospitalization (2). A probiotic protocol similar to what was used in a prospective intervention study at one large skilled-nursing home may be considered (14). A 3-strained Lactobacilli blend was started (2 capsules of Bio-K+ once daily) within 24 h of the antibiotic start and continued while the patient was on the antibiotic. After the antibiotics were stopped, the probiotic was continued for an additional 7 days. A significant reduction of CDI rates were noted at this facility after the probiotic blend was implemented. Contraindications for probiotic use include severely immunocompromised states or patients at high risk for translocation, which can lead to sepsis. Dentists should be aware of which probiotic products have evidence-based clinical studies so they can educate their patients and help inform them of signs and symptoms of AAD and CDI.

In conclusion, not all probiotic products are equal. Evidence-based studies show usefulness of some probiotics, but not all for the prevention of AAD and CDI. Until further guidance is available, practitioners might consider adding one of the following probiotics during antibiotic administration and for 5–7 days after antibiotics are discontinued to allow the oral microbiome to recover: *S. boulardii* CNCM I-745 (Florastor™), *L. rhamnosus* GG (Culturelle™) or the 3-strain blend of *L. acidophilus* CL1285, *L. casei* LBC80R and *L. rhamnosus* CLR2 (Bio-K+ ™). The role of probiotic strains to reduce dental caries and treat periodontal disease requires more research. The role of probiotics in dentistry requires further studies.
